# The promise and potential challenges of intermittent preventive treatment for malaria in infants (IPTi)

**DOI:** 10.1186/1475-2875-4-33

**Published:** 2005-07-20

**Authors:** Wendy Prudhomme O'Meara, Joel G Breman, F Ellis McKenzie

**Affiliations:** 1 Division of Epidemiology and Population Studies, Fogarty International Center, National Institutes of Health, Bethesda, MD 20892 USA

## Abstract

Intermittent preventive treatment (IPT) administers a full therapeutic course of an anti-malarial drug at predetermined intervals, regardless of infection or disease status. It is recommended by the World Health Organization (WHO) for protecting pregnant women from the adverse effects of malaria (IPTp) and shows great potential as a strategy for reducing illness from malaria during infancy (IPTi). Administered concurrently with standard immunizations, IPTi is expected to reduce the frequency of clinical disease, but to allow blood-stage infections to occur between treatments, thus allowing parasite-specific immunity to develop. While wide deployment of IPTi is being considered, it is important to assess other potential effects. Transmission conditions, drug choice and administration schedule will likely affect the possibility of post-treatment rebound in child morbidity and mortality and the increased spread of parasite drug resistance and should be considered when implementing IPTi.

## Background

**Figure 1:**
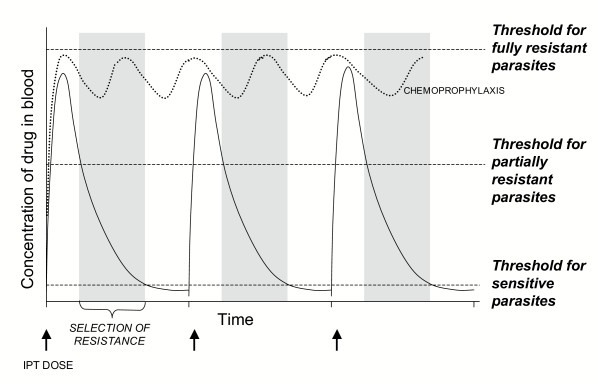
**Concentration of anti-malarial drug in bloodstream during IPT. **Drug concentration initially rises after administration of a therapeutic drug regimen. The concentration declines gradually as the drug is cleared from the bloodstream. Parasites with increasing sensitivity to the drug will be able to grow as the drug concentration declines. Parasites are divided roughly into three categories; fully sensitive, partially resistant and fully resistant. The interval between the time at which the concentration falls below the threshold for partially sensitive parasites and the time at which it falls below the threshold for sensitive parasites is a window of selection for resistant parasites. The dotted curve represents the target blood concentrations during chemoprophylaxis.

Intermittent preventive treatment (IPT) protects pregnant women from malaria and placental parasitaemia and their newborns from malaria-associated low birth weight [1-6]. This strategy is being proposed to reduce malaria morbidity and mortality in infants (IPTi) in sub-saharan Africa by administration of a therapeutic course of an anti-malarial drug at predetermined intervals, regardless of infection, during routine vaccinations of infants in the Expanded Programme on Immunization (EPI). The intention is to clear any current infection and protect against new infection until the drug level decays in the bloodstream. Intervals between doses are typically longer than the time to clear the drug from the bloodstream (Figure 1), allowing the possibility of infection between doses. In infants, IPTi may simultaneously reduce the frequency of infection and life-threatening disease while allowing immunity to develop.

The context of IPTi is fundamentally distinct from that of IPTp: recipients of IPTp have already acquired some parasite-specific immunity, whereas infants are immunologically naïve and may comprise a disproportionately large infectious reservoir in the community [7-9]. A theoretical evaluation of the effect of drug pharmacokinetics on IPTi and IPTp efficacy in the face of rising drug resistance has recently been presented [10]. This paper discusses concerns related primarily to IPTi, specifically, whether: 1) immunity developed to malaria will be as robust in treated as in untreated individuals, forestalling a post-treatment increase in disease and mortality (rebound effect), 2) differences in transmission intensity should inform the timing of IPTi administration, 3) IPTi is likely to promote the spread of drug resistance, and 4) regional differences should inform the choice of IPTi drug.

## Published IPTi studies

Two published studies have examined the effect of IPTi on malaria morbidity. Both were conducted in Tanzania and showed significant decreases in anaemia and clinical episodes of malaria in the treated group relative to the placebo group. In the study with sulfadoxine-pyrimethamine (SP), infants were given either a placebo or a drug three times with vaccinations (at two, three and nine months of age) and were followed until 22 months of age [11]. IPTi reduced the expected number of clinical cases of malaria in the first year of life by 59%. However, the effect declined significantly with time after the final dose. One month after the last dose – roughly the time to clear the final dose of SP from the bloodstream – the clinical case rates among infants in the control group and those in the treated group who had not previously contracted clinical malaria were indistinguishable and remained so until the end of the follow-up period. The follow-up to this study [12] reported that the protective effect of IPTi with SP extended well into the second year of life; however, only those children who contracted malaria during IPTi treatments experienced this long-term benefit. Those who did not contract malaria during IPTi treatments had malaria as frequently as placebo recipients who did not contract malaria. In the study with amodiaquine [13], infants three to four months old were given a full course of the drug every two months for six months and followed for four months after the last dose. The protective efficacy against clinical attacks of malaria was 65% during the six months of treatment. Some protection persisted throughout the follow-up period; by the end of the study, 35% of treated infants remained free from a first attack of malaria compared to 13% in the control group.

Thus, the results of the two published IPTi studies are encouraging. Two other studies [14,15] using strategies similar to IPTi failed to demonstrate statistically significant reductions in clinical episodes of malaria. Both administered SP at monthly intervals three times to anaemic children in Kenya. These children were older than those in the two IPTi studies (mean age at enrollment = 11 [14] and 20 months [15], versus 0 [11] and three [13] months), but still within the age range most at risk for malaria-related morbidity and mortality in these regions [16].

## Immunity and rebound

An effective immune response to malaria is acquired slowly and cumulatively, requiring boosting; each subsequent infection generates increasing protection from severe illness, although the exact number of infections required to achieve a protected state is unknown. Of concern is whether administering anti-malarial drugs interrupts development of immunity and leads to decreased malaria-specific immunity and/or a rebound of increased morbidity and mortality after termination of treatment.

Rebound effects have been observed in several studies of chemoprophylaxis in children [17-21]. Using pyrimethamine-dapsone (P-D), Menendez *et al *[17] found that children given weekly doses during their first year had fewer malaria episodes during that year than did controls; in the year after termination of chemoprophylaxis, the frequency of cases in the treated group was twice that of the placebo group (Table 1). In a longer-term study, children were given P-D or placebo every two weeks from three months to five years of age or until the end of the five-year study, whichever was first [18,22]; at its conclusion, the study included children between one and ten years of age who had been treated for up to five years. The number of malaria episodes in the post-treatment group was greater than in the placebo group, and the probability of death from malaria between the age of five and six, immediately after termination of treatment, was slightly higher in the group that had received P-D for the full five years than in the placebo group. However, there was a 15% overall reduction in mortality in the treated group from age 0 to 10 years. Geerligs *et al *[23] review malaria chemoprophylaxis in children, including studies with and without rebound effects. Von Seidlein and Greenwood [24] offer a more general review of mass drug administration strategies including chemoprophylaxis and IPT.

**Table 1:** Comparison of studies during which a rebound effect was observed upon termination of drug treatment. Studies are in the order they are cited in the text.

**Drug used (country)**	**Ages of study group**	**Duration of treatment**	**Duration of post-intervention follow-up**	Effect on malaria morbidity	**Rebound effect**	**Reference**
P-D^1 ^(Tanzania)	2 mo. at start of study	Weekly for one year	One year after termination of treatment	Reduced incidence of clinical malaria by 40% during treatment period	80% higher incidence of clinical episodes in treated group during the year following termination of treatment	[17]
P-D (The Gambia)	3 mo. at start of study	Every 2 weeks for maximum of 5 years	5 years	65% reduction in malaria episodes after 3 years of chemoprophylaxis	52% more cases in treated group during the year following termination of treatment	[18, 22]
SP^2 ^+ artesunate (The Gambia)	Entire villages, all ages	MDA^3 ^1 dose	20 weeks	Reduced rate of malaria attacks in children <11 yr by 60%	Rate of clinical malaria was 69% higher in treated groups 3 months after treatment	[25]
SP (Mali)	3 mo. to 20 years	MDA 1 dose	24 weeks	Reduced incidence of first malaria episode from 26% to 3% during first month	Incidence of first malaria episodes in treated group rose to 42% compared to 17% (untreated group) during the third month after treatment	[26]

^1^P-D: pyrimethamine-dapsone

^2^SP: sulfadoxine-pyrimethamine

^3^MDA: mass drug administration

Among the implicit assumptions of the IPTi strategy are that both the number of serious clinical episodes and the development of malaria-specific immunity are directly proportional to the frequency of infection. If these assumptions are true, intermittent doses of drug could reduce the frequency of serious malaria episodes, while the intervals between doses of drug could permit infection and the development of immunity. Thus, if suppressed development of immunity is responsible for any rebound effects from chemoprophylaxis, the IPTi strategy could reduce the likelihood of rebound, provided that infectious bites occur frequently enough to ensure exposure between doses. There is reason to doubt whether rebound effects are proportional to the frequency of infection and immunity in infants and children, however. In the five-year study with P-D cited above, the group that had received the drug for the full five years had levels of malaria-specific antibodies equivalent to those in the control group [18]. Nonetheless, they experienced more disease episodes and mortality during the first year post-chemoprophylaxis than those with lower antibody titres. Parasite-specific antibody titres often correlate with exposure, but not protection from disease.

The relationship between frequency of infection, immunity and rebound is further complicated by evidence that very short-term drug administration can lead to significant rebound effects (Table 1). During a one-time mass administration of SP-plus-artesunate to entire villages in The Gambia at the start of the rainy season [25], the frequency of malaria attacks among children under 11 years of age in treated villages was 60% lower than in control villages during the first six weeks. During the third month post-treatment, disease incidence doubled in children in the treated villages relative to the placebo villages. In another study [26], two groups of individuals from the same town in Mali, aged three months to 20 years, were treated with a single dose of SP or placebo. In the first four weeks, 5% of the treated group had at least one malaria episode compared to 25% of the control group. During weeks nine to 12, the incidence was twice as high in the SP group. These last examples included much wider age ranges than would IPTi, but they indicate that rebound can happen over very short time periods, with only a single dose of drug, signaling the possibility that IPTi could show similar effects.

Neither of the published IPTi studies reported a rebound after termination of treatment, even after extended follow-up. However, a significant rebound of malaria morbidity was observed in an IPTi trial conducted in Navrongo, Ghana (D. Chandramohan, personal communication). In this study, the number of rebound episodes apparently did not negate the benefit of IPTi. Even so, it is imperative that these effects be measured and reported prior to community-wide implementation of IPTi.

## Transmission effects: timing of IPTi administration

It is important that the conditions for which the current conception of IPTi within the EPI delivery system will be beneficial be defined as clearly as possible so that resources for malaria control are allocated to the most effective programmes in each region. Under conditions of intense, perennial transmission, infants may be susceptible to severe clinical attacks from a few months after birth, and antimalarials given during routine EPI visits at two, three and nine months of age are likely to be effective in reducing episodes of malaria. Where transmission is less intense or unstable, children are at great risk until 5 years of age or older. As transmission rates decline with increasing urbanization and bednet usage in sub-Saharan Africa [27,28], a larger proportion of the population will be at greatest risk after one year of age. For example, the average age of first malaria infection in Prampram, Ghana [29], was estimated to be 10.5 months; here, IPTi within EPI (final dose at nine months) is unlikely to impact malaria morbidity. Thus, implementing IPTi uniformly and exclusively at the time of EPI vaccinations may be inadequate in many circumstances.

**Figure 2:**
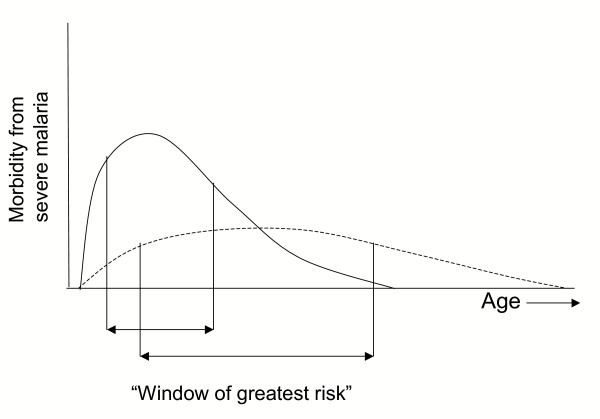
**Effect of transmission intensity on outcome of IPTi. **Qualitative depiction of the effect of transmission intensity on the age-incidence pattern of malaria morbidity and mortality. In areas of high transmission (solid line), malaria mortality is concentrated in the first two years of life. In areas of low or unstable transmission (dashed line), children may be at risk until much later in life. The arrows represent the *window of greatest risk *or the age interval during which roughly 75% of childhood malaria episodes are experienced.

If the aim is to administer IPTi during the time of greatest risk of severe disease from unprotected exposure to infection, age-incidence data for severe malaria will be needed to target those most likely to benefit in a given location (Figure 2). In low and moderate transmission areas, IPT may be most effective if the schedule is extended into early childhood. Highly seasonal malaria may require unique strategies which target the appropriate age groups during the transmission season. If mechanisms for delivery can be established that allow greater flexibility in the window of administration, IPTi could have a much broader impact. A study of IPT in children under five in an area of highly seasonal transmission administered doses of SP at monthly intervals exclusively during the high transmission season. This approach reduced the incidence of clinical malaria by more than 80% in the treated group compared to the control group, indicating that adapting the IPT schedule can be accomplished with good results [30].

The relationship between the age interval during which IPTi is administered and that of greatest risk will determine the overall benefit of IPTi, i.e. reduction in the total number of malaria cases. In the published SP study [11], prevalence among 12-month old infants was only 4% (annual Entomological Inoculation Rate = 29 [31]) whereas in the amodiaquine study the prevalence in 3.5 month-olds was 24–34% (EIR = 400). Although protective efficacy, or the percentage reduction in first clinical episodes, was similar between the two studies (59% and 65%), only 0.26 cases per person-year at risk were prevented in the SP study versus 1.49 cases in the amodiaquine study.

**Figure 3:**
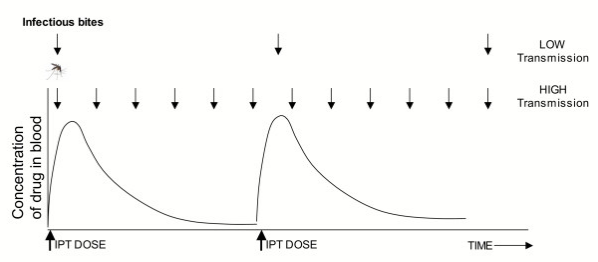
**Effect of transmission intensity on outcome of IPTi. **Effect of biting frequency (arrows represent infectious bites) on the development of malaria-specific immunity during IPTi. The frequency of infectious bites determines whether or not an infant is exposed to malaria parasites between doses of drug during IPTi.

The frequency and clustering of infectious bites determines whether the time interval between doses will permit the development of immunity, and whether IPTi will act primarily to protect against infection or treat existing infections (Figure 3). Exposure to infectious bites must be sufficiently frequent, or IPT doses sufficiently spaced, to allow infection between periods of protection. Although the relationship between infection and development of clinical immunity remains poorly understood, it is clear that if no infectious bites are received between treatments, then IPTi will be functionally equivalent to chemoprophylaxis.

Transmission intensity may vary considerably over very short distances and short time periods. While it will often be impractical to vary drug administration schedules in response, broader assessments of local transmission and disease patterns should guide IPTi schedules.

## Drug resistance

IPTi has the potential to both affect and be affected by parasite resistance to antimalarial drugs. The possible relationship between drug resistance and the efficacy of IPTi has been discussed elsewhere [10]. Paradoxically, truncating an infection may interrupt the development of protective immunity leading to speculation about whether drugs which do not completely clear an infection may be more effective for IPTi. Data about frequencies of resistance at or near IPTi study sites (see below) will shed light on this relationship. The impact of IPTi on the spread of drug resistance will be difficult to assess, particularly where the study group is a small fraction of the community and the drug studied is also used for treatment of malaria episodes.

### Drug pharmacokinetics

**Figure 4:**
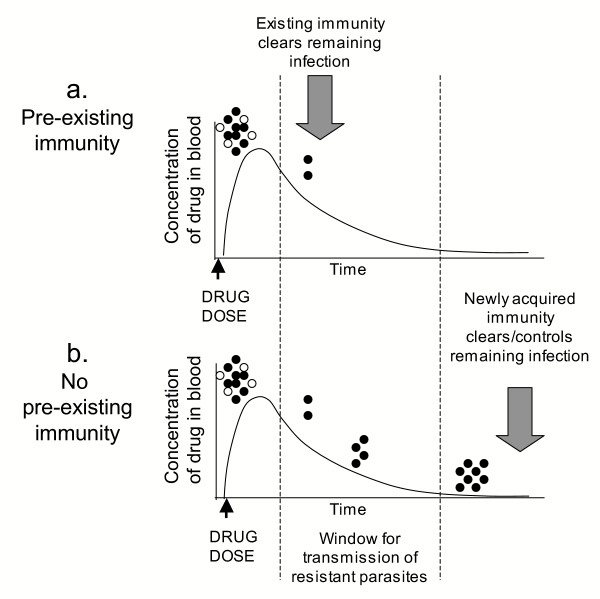
**Drug treatment and immune mechanisms act synergistically to eliminate parasites. (a) **A patient with some pre-existing immunity clears infection and reduces the number and transmission of the parasites that survive treatment. (b) Without pre-existing immunity, the window for proliferation and differential transmission of resistant parasites is extended. White and black circles represent sensitive and resistant parasites, respectively.

After administration of an anti-malarial drug, the concentration in the bloodstream initially peaks and then gradually declines. During successful treatment, parasites are damaged or killed by the drug and cleared by the immune system. An infection may include a mixture of genetically heterogeneous parasites with varying drug sensitivities. Drug-sensitive parasites are completely cleared by drug treatment and cannot grow in the presence of low concentrations of the drug. Fully resistant parasites survive "therapeutic" doses of a drug, whereas partially resistant parasites can grow in the presence of a drug, but only when the concentration drops below a critical threshold level (Figure 1). While the concentration remains above the critical threshold for sensitive parasites, resistant parasites are selected and competition from sensitive parasites is removed [32]. For instance, Watkins *et al *[33] showed that infections detected during the clearance of SP, but before SP concentration drops to zero (15–52 days post-treatment), are more likely to be pyrimethamine resistant. The spread of drug resistance is promoted by increased transmission of resistant strains relative to sensitive strains in the interval following drug treatment and before the immune system succeeds in clearing parasites [34] (Figure 4a).

During chemoprophylaxis, the intention is to administer the drug often enough to prevent plasma concentrations from falling below the survival threshold for resistant parasites (Figure 1). Lack of compliance resulting in fluctuations in drug concentrations increases parasite exposure to sub-preventive/sub-curative levels and promotes the development and spread of drug-resistant parasites. Compared to chemoprophylaxis, IPTi offers the advantage of decreased drug pressure through supervised doses that target a specific, relatively small age group. It has the disadvantage of causing cyclical fluctuations of drug titres ideal for repeated selection of resistant parasites.

IPTi studies have chosen drugs with long half-lives to maximize the prophylactic window of each dose. However, long clearance times also lead to long periods during which selective levels of drug are present in the bloodstream, and, when these drugs are administered to infants, the parasites are exposed to selective concentrations in an immunologically naive individual. With little or no pre-existing immunity, the host is less able to control an infection, so the potential for growth and transmission of resistant parasites is enhanced (Figure 4b). Preliminary results from two studies with SP in children under five years of age show increased frequencies of infections with SP-resistant parasites among children receiving IPT [30,35]. It is not known how much this increase will increase the community-wide transmission and prevalence of drug resistance, but several studies indicate that infants and young children are the most important infectious reservoir in a population, despite their minority [7-9]. Prospective, community-wide, cross-sectional surveys of molecular markers of parasite resistance before and after implementation of IPTi could address this question. Of course, in order to differentiate between selective pressure due to conventional treatment of malaria in the community and that due to IPTi, a drug not routinely given for febrile episodes must be chosen for IPTi in such a study.

### Genetics of resistance

In infants, chemoprophylaxis decreases the multiplicity of infections (MOI), the number of distinguishable genotypes based on a single locus, present at any time in a single host [36]. Assuming proportional transmission, with fewer genotypes per host there is less potential for mating between parasites with different alleles at resistance loci. If resistance is conferred by alleles at two or more loci, inbreeding reduces the opportunity for the resistance alleles to be separated, and thus the probability that offspring will be susceptible. Parasite inbreeding increases the survival and prevalence of resistance genes [34].

**Figure 5:**
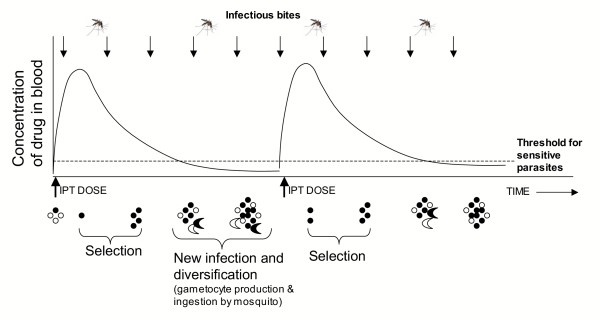
**Effect of IPT on parasite diversity. **Blood concentration of drug during two treatments of IPT is shown. All of the sensitive parasites (white circles) initially present in the host at the start of IPT are killed by the drug, leaving only resistant parasites (black circles). While the drug concentration is high, new infections are prevented and the resistant parasites proliferate without competition. When the drug concentration falls below the threshold for sensitive parasites, an infectious bite can produce an infection with sensitive parasites. A subsequent bloodmeal may take up gametocytes from both sensitive (white crescents) and resistant strains (black crescents), thereby allowing out-crossing between them. At the next treatment, the sensitive parasites are killed and the resistant parasites continue to proliferate and may acquire new genetic material at the next cycle. Black arrows at the top represent continual biting by infectious mosquitoes.

Alternatively, IPT could increase antigenic diversification of drug-resistant strains (Figure 5). A treatment dose will kill all the sensitive parasites but may leave a few resistant parasites to multiply as the drug concentration declines. When the drug is completely cleared, the host becomes vulnerable to infection by drug-sensitive parasites and so may acquire multiple strains which can cross with resistant parasites. The total effect is to select for resistant parasites, allow them to multiply without competition and provide material for antigenic diversity in the next generation, via cross-breeding in the mosquito, before administering the drug again and once more selecting for the resistant parasites.

It is not known how IPTi would affect the dynamics of multi-strain infections, or whether IPTi would increase parasite inbreeding, and the prevalence of resistance genes, or increase out-crossing and the antigenic diversity of resistant populations. Transmission intensity will determine which, if any, of these outcomes is observed. Because the spread of resistant parasites could be significantly affected in either case, these potential outcomes of IPTi should be considered in designing implementation schemes.

## Drug choice

Whether or not a particular drug is effective may be determined by the mechanism of protection of IPTi. If the prophylactic period is the primary protective mechanism, then drugs which are cleared very quickly, such as artemisinin, are not likely to be useful, either alone or in combination with other drugs. If the primary mechanism is clearance of existing infections, then drugs with very short half-lives should be very effective and would decrease the probability of accelerating the spread of resistance. Studies in mice have shown that suppression of blood-stage infection enhances development of protective immunity against liver-stage infection [37,38]. If the same is true in human hosts and if infectious bites occur between doses of drug, IPTi could protect by enhancing liver-stage immunity. In this case, drugs active against the liver stage of the parasite would not be effective. Several drug candidates with both long and intermediate persistence times, such as chlorproguanil/dapsone (Lapdap), SP with artesunate, amodiaquine with artesunate and mefloquine, are being tested in ongoing IPTi trials .

When choosing from among the effective drug candidates, the context in which IPTi will be implemented should also be considered, including the regional level of resistance to drugs available for IPTi and the community use of drugs for treatment of symptomatic malaria. If infants who present with clinical malaria during IPTi are treated with the same drug that is used for IPTi, the spread of drug-resistant parasites may be accelerated: an infection that appears during IPTi is more likely to be resistant to some level of that drug, so dual usage could lead to an increased selection and spread of highly resistant parasites.

The studies which will be completed in the near-term are using SP and it seems likely that SP will be the drug chosen for initial implementation of IPTi. However, an anti-folate drug closely related to SP, cotrimoxazole (trimethoprim-sulfamethoxazole), is currently recommended for prophylaxis against opportunistic infections in HIV-positive individuals [39] and is widely used in infants and children in malaria-endemic areas where IPTi could be implemented. Cross-resistance to SP and cotrimoxazole has been reported in *Plasmodium falciparum *[40] and may have serious consequences for malaria-infected patients undergoing chemoprophylaxis with cotrimoxazole. Where there is a high prevalence of HIV among infants at risk for malaria, the effect of cross-resistance to cotrimoxazole on efficacy of IPTi with SP must be evaluated and vice-versa.

## Studies underway

There are several clinical trials of IPTi underway, six of which are being conducted within the IPTi Consortium . The annual EIRs among these six sites range from five to 200 infectious bites per person, allowing comparison of efficacy data from a relatively broad range of transmission conditions. However, if IPTi is uniformly administered at two, three and nine months of age, no information about the broader applicability of IPTi will be available from these studies. For example, if IPTi delivered through EPI offers no significant protection where the EIR is less than 50, it will be desirable to know if different schedules might be effective. New studies would need to be designed to answer this question.

Since an increase in drug resistant infections [30,35] and a rebound in malaria episodes (D. Chandramohan, personal communication) have been observed in IPTi studies, these possible adverse outcomes should be measured in further studies. Concerns have also been raised about the effects of IPTi on post-vaccination seroconversion [41]. If markers of immunity, EPI seroconversion and drug resistance data can be integrated in a meta analysis across these diverse trials, this would provide considerable evidence for evaluating the safety of this scheme of IPTi implementation. This effort would require extensive cooperation and standardized methods, but the result would be an unprecedented ability to effectively compare an intervention across trials in diverse malaria transmission zones. Information available online  indicates that the IPTi Consortium is coordinating its cost-effectiveness and drug safety studies in precisely this manner.

## Conclusion

IPTi is a promising new intervention strategy which aims to combine the short-term protective mechanisms of chemoprophylaxis with the long-term protection of naturally acquired immunity. It is certain that for some age interval, over some time interval, IPTi would reduce the clinical malaria case rate, whether the drug is acting as prophylaxis, or to clear sub-clinical infections, or both. However, the price to be paid in the broader community, for example in immunity and drug resistance, will be greatly affected by how well IPTi deployment addresses the local context of transmission and drug efficacy. While it is likely that IPTi linked to EPI immunization will be salutary in areas with intense stable transmission, much of Africa is moving toward reduced or unstable endemicity through urbanization and use of insecticide impregnated bednets and other vector control strategies. If bednet use prevents infections during the period of IPTi treatment (i.e. the first year of life), then IPTi will not add protection. Furthermore, if, in fact, the extended protective effect of IPTi depends on becoming infected during that period, IPTi programmes in areas of high bednet usage may not show the expected effects on malaria morbidity. The efficacy and practicality of IPTi under different schedules of administration should be explored.

To help verify that the intention of IPTi is realized, the effects of IPTi on malaria-specific acquired immunity should be measured and follow-up periods should include sufficient time for rebound to be evaluated. More thorough assessment of the potential impact of IPTi on the spread of drug resistance is also necessary. Amplification of drug resistance may be exacerbated by special factors connected with regimented, symptom-blind drug use in immunologically naive infants. Site-sensitive choices of drug and scheduling would help to minimize the amplification and maximize the chance that it will eventually be counter-balanced by decreased drug use later in life.

## Authors' contributions

The manuscript was written by W.P.O. All authors contributed to the concepts and ideas presented and to the editing of the final manuscript.

## Conflict of interest statement

The author(s) declare that they have no competing interests.
